# Pulmonary Collision Tumor Comprising Lung Adenocarcinoma and Metastatic Endometrial Cancer: A Case Report

**DOI:** 10.1111/1759-7714.70339

**Published:** 2026-07-04

**Authors:** Momoko Asami, Hayato Konno, Shinya Katsumata, Hideaki Kojima, Naoya Yokomakura, Mitsuhiro Isaka, Nobuhiro Kado, Takuya Kawata, Masahiro Endo, Yasuhisa Ohde

**Affiliations:** ^1^ Division of Thoracic Surgery Shizuoka Cancer Center Shizuoka Japan; ^2^ Division of Gynecology Shizuoka Cancer Center Shizuoka Japan; ^3^ Division of Pathology Shizuoka Cancer Center Shizuoka Japan; ^4^ Division of Diagnostic Radiology Shizuoka Cancer Center Shizuoka Japan

**Keywords:** collision tumor, endometrial cancer, lung adenocarcinoma

## Abstract

We report a rare case of a 78‐year‐old woman with a collision tumor composed of primary lung adenocarcinoma and metastatic endometrial cancer. The patient previously underwent hysterectomy and adjuvant chemotherapy for Stage IIIC1 endometrial cancer. Chest computed tomography (CT) revealed a ground‐glass nodule in the right upper lobe, which was suspected to be inflammatory scarring. Two years later, new adjacent nodules emerged, both of which gradually increased in size and exhibited partial collisions. A lobectomy was performed to detect multiple synchronous lung cancers. Histological features of the surgical specimens included acinar adenocarcinoma and metastatic lung tumors of endometrial cancer. To the best of our knowledge, this is the first report of a pulmonary collision tumor consisting of a lung adenocarcinoma and a metastatic lung tumor of endometrial cancer, with serial CT imaging successfully capturing the collision between the two lesions.

## Graphical abstract

A rare pulmonary collision tumor comprising primary lung adenocarcinoma and metastatic endometrial cancer was identified within a growing part‐solid nodule. Histopathology confirmed two distinct colliding tumor components.

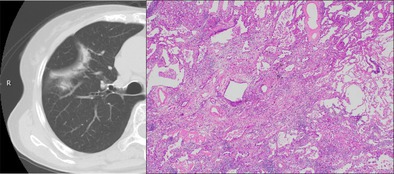

## 1 Introduction

Collision tumors represent a rare subset of synchronous multiple primary malignancies characterized by the close apposition or partial intermingling of two histologically distinct neoplastic entities within the same anatomical site. Herein, we report a case of a pulmonary collision tumor comprising primary lung adenocarcinoma and a metastatic lung lesion originating from endometrial cancer.

## 2 Case Presentation

A 78‐year‐old woman underwent hysterectomy following the diagnosis of endometrial cancer and subsequently received postoperative adjuvant chemotherapy for pT2N1M0 Stage IIIC1 disease. The patient had no history of smoking or malignancies other than endometrial cancer. At the time of the endometrial cancer diagnosis, chest computed tomography (CT) revealed a ground‐glass nodule in the upper lobe of the right lung (the first lesion). The nodule exhibited contractile changes and was initially considered to be an inflammatory scar. Consequently, the patient underwent surveillance with chest CT every 6 months.

**FIGURE 1:**
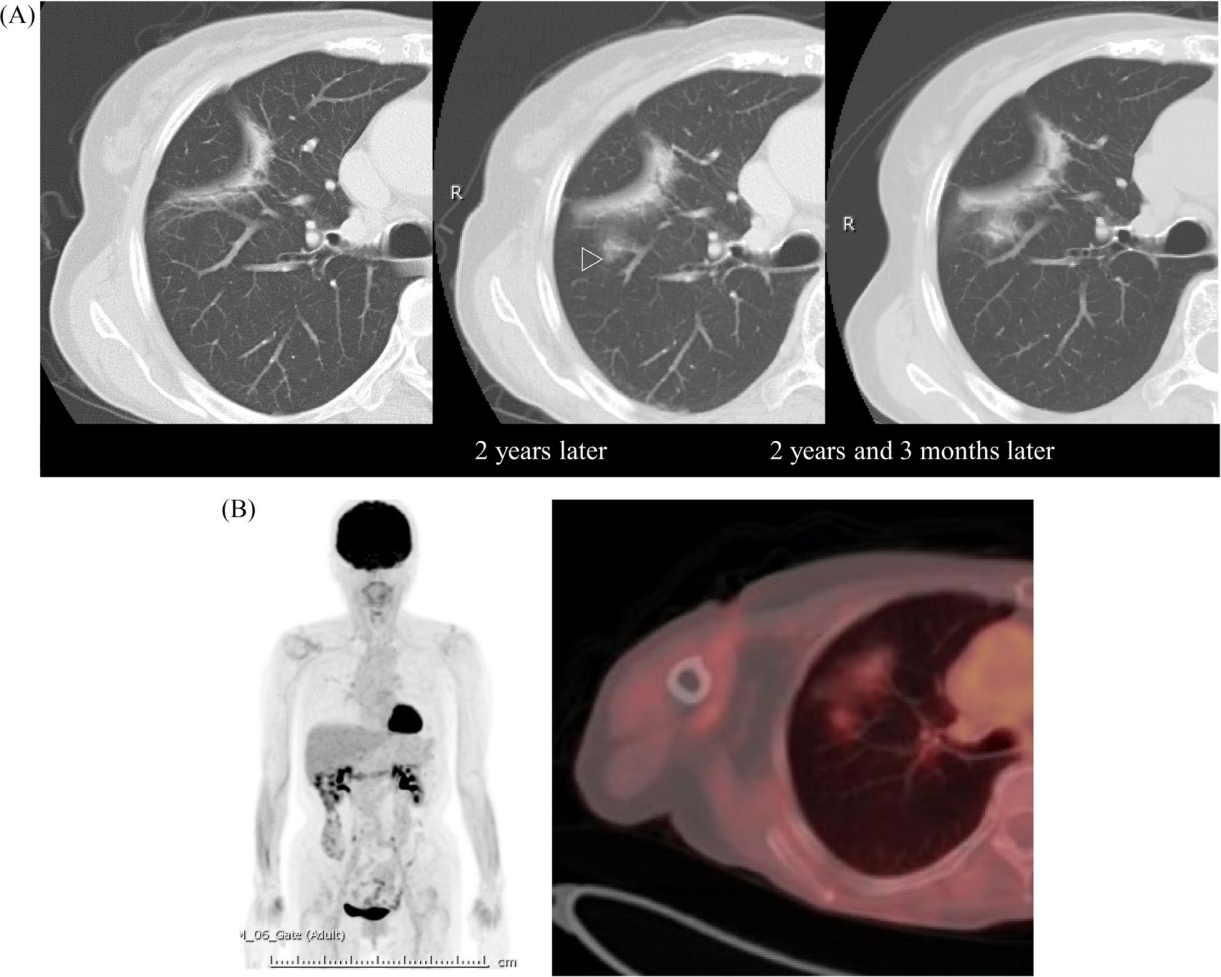
(A) Chest CT scan showing the appearance of a second lesion (▷) adjacent to the first lesion in the right upper lobe. Over time, both nodules increased in size and eventually merged, forming an area of radiological fusion. (B) PET‐CT demonstrating low fluorodeoxyglucose (FDG) uptake with SUV_max values of 1.6 for the first lesion and 1.79 for the second lesion.

Two years after surgery for endometrial cancer, follow‐up CT revealed the emergence of a new ground‐glass nodule (second lesion) adjacent to the first lesion. Both nodules gradually increased in size during the observation period and partially collided (Figure 1). The first lesion appeared as a solid lesion with relatively well‐defined margins on chest CT. In contrast, the second lesion demonstrated a faint ground‐glass opacity with slightly indistinct margins. During the follow‐up period, the ground‐glass nodule showed interval changes, including a gradual increase in internal attenuation while maintaining a predominantly ground‐glass appearance. Whole‐body positron emission tomography (PET)‐CT revealed standardized uptake values (SUVmax) of 1.6 in the first lesion and 1.79 in the second lesion (Figure 1). On the basis of these findings, multiple synchronous lung cancers were suspected. The clinical stages of the tumors were determined to be T3N0M0‐Stage IIB and T1bN0M0‐Stage IA2, respectively.

**FIGURE 2:**
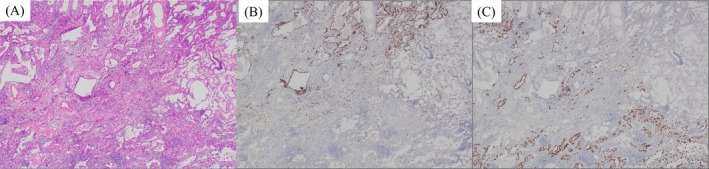
(A) Microscopic findings showing direct collision between two histologically distinct tumor components, particularly in the central area of the lesion. The collision interface is considered to correspond to the area of radiological fusion observed between the two nodules on preoperative CT imaging (B) Immunohistochemical staining of the adenocarcinoma component showing TTF‐1 positivity. (C) Immunohistochemical staining of the metastatic clear cell carcinoma component showing PAX‐8 positivity.

The patient underwent right upper lobectomy with systematic mediastinal lymph node dissection. Histopathological examination of the resected specimen revealed two distinct neoplastic entities. The first lesion, which was initially considered an inflammatory scar, predominantly exhibited an acinar growth pattern with extensive areas of collapsed fibrosis beneath the pleura. Immunohistochemical analysis revealed positivity for thyroid transcription factor‐1 (TTF‐1), confirming the diagnosis of primary lung adenocarcinoma. The patient was diagnosed as pathologically T2bN0M0‐ stage IIA. The second newly appearing lesion was composed of highly atypical cells with a clear cytoplasm and a high nuclear‐to‐cytoplasmic ratio, demonstrating a replacement growth pattern. Immunohistochemical staining revealed positivity for hepatocyte nuclear factor 1‐beta (HNF1β) and paired box gene 8 (PAX‐8), with a lack of TTF‐1 expression, leading to the diagnosis of metastatic lung involvement from previously diagnosed endometrial cancer. In addition, metastatic involvement of the hilar lymph nodes (stations 11s and 12u) was observed. These metastatic lymph nodes were positive for PAX‐8 and negative for TTF‐1, further supporting lymph node metastasis from endometrial cancer. Microscopic examination revealed a direct continuity between the two lesions, consistent with the diagnosis of a collision tumor. Histopathological analysis revealed the collision of two distinct neoplastic components, acinar adenocarcinoma and metastatic clear‐cell carcinoma (Figure 2).

The postoperative course was uneventful. Following lung resection, the patient was considered ineligible for adjuvant chemotherapy and was therefore managed with close postoperative surveillance. Adjuvant chemotherapy was not administered for the lung adenocarcinoma component, considering the patient's age and the clinical context of the previously treated endometrial cancer. For the endometrial carcinoma component, no additional systemic therapy was indicated, given the lack of established evidence supporting adjuvant chemotherapy after complete resection of localized recurrent disease and the absence of microsatellite instability‐high status. Peritoneal dissemination of endometrial carcinoma was detected 14 months after lung resection, and the patient received systemic chemotherapy. She remains alive without evidence of lung cancer recurrence.

## 3 Discussion

This case represents a rare instance of a pulmonary collision tumor involving primary lung adenocarcinoma and a metastatic lung tumor from endometrial cancer, a combination that has not been previously reported. Additionally, preoperative CT imaging confirmed gradual enlargement and eventual merging of the tumors over time, a rarely documented feature. Most reported cases of collision tumors are diagnosed incidentally through histopathological examination, which makes preoperative recognition extremely challenging. In the present case, the possibility of a collision tumor was not suspected based on the preoperative imaging findings, and the diagnosis was established only after the final pathological examination. These findings highlight the limitations of imaging in detecting collision tumors and underscore the importance of histopathological evaluation.

Collision tumors, first proposed by Meyer in 1919 [1], are defined as independent neoplasms composed of two or more histologically distinct tumor components that coexist within the same anatomical site, grow in close proximity, and remain sharply demarcated without histologic intermingling [2]. The diagnostic criteria include the following: (1) two distinct histological types that can be clearly differentiated; (2) each tumor retaining its own histological characteristics even in adjacent areas; and (3) although partial intermingling or transitional zones may occasionally be observed at the interface (collision site), the two components remain fundamentally distinct [3]. These criteria allow distinction from mixed tumors, in which different histological components are intermingled within a single neoplasm.

The exact mechanism underlying collision tumors remains unclear, but several theories have been proposed: random collision effect—collision tumors may arise by chance when two independent malignancies coexist in the same organ [4, 5]. Field Cancerization: Genetic alterations caused by carcinogenic events may lead to the development of multiple tumors within the same tissue [6, 7]. Tumor‐to‐Tumor Metastasis: One tumor may serve as a host for cells metastasizing from another, forming a collision tumor [8, 9]. Tumor Microenvironment Effect: The first tumor may modify the local microenvironment, facilitating the growth of a nearby second tumor [10, 11]. In our case, the primary lung adenocarcinoma and metastatic endometrial cancer likely developed independently and expanded until they collided. Lung adenocarcinoma may alter the microenvironment, promoting adjacent growth of the metastatic tumor.

Among the reported cases, only a few demonstrated tumor collisions over time using serial chest CT imaging [12]. In most cases, collision tumors are incidentally diagnosed based on the histopathological examination of surgical specimens. The preoperative identification of collision tumors remains challenging. In previously reported cases where tumor collisions were identified on chest CT, lesions typically appeared as solid nodules. To our knowledge, this is the first report of a ground‐glass nodule that gradually enlarged and eventually coalesced over time. Notably, no imaging features were specific to collision tumors.

To date, six cases of collision tumors involving primary and metastatic lung tumors have been reported (Table 1) [13, 14, 15, 16]. Breast cancer, adenoid cystic carcinoma, and choriocarcinoma have been reported to be the origins of metastatic lung tumors. There have been no previous reports of lung metastases from endometrial cancer. In all the cases, the diagnosis of a collision tumor was not preoperatively predicted. In our case, chest CT revealed gradual enlargement and eventual merging of the two nodules over time. Notably, both primary lung cancer and metastatic lung tumors presented as ground‐glass nodules, which are atypical for metastatic lung tumors. Pulmonary metastases are typically observed as solid nodules; however, atypical presentations such as ground‐glass opacities have been reported. Lepidic growth of metastatic tumor cells along the alveolar walls has been described as one possible mechanism underlying such findings, resulting in an air‐space pattern on CT imaging [17]. In the present case, the gradual increase in internal attenuation while maintaining a predominantly ground‐glass appearance may reflect this growth pattern.

**TABLE 1:** Clinical cases of pulmonary collision tumor consisting of primary lung cancer and metastatic lung tumors described in the literature.

Case	Patient age/Sex	Primary lung cancer	Metastatic tumor	Preoperative CT confirmed collision findings
Lin [13]	55/F	Adenocarcinoma	Adenoid cystic carcinoma (Maxillary Sinus)	No
Piacentini [14]	75/F	Squamous cell carcinoma	Ductal breast carcinoma	No
Piacentini [14]	59/F	Adenocarcinoma	Lobular breast carcinoma	No
Blanco [15]	56/M	Adenocarcinoma	Adenoid cystic carcinoma (Maxillary Sinus)	No
Wang [16]	55/F	Squamous cell carcinoma	Choriocarcinoma	No
Present case	78/F	Adenocarcinoma	Endometrial cancer	Yes

Collision tumors are rare entities, and their prognostic implications remain unclear because of the limited number of reported cases. In general, the clinical outcome is considered to depend not on the collision phenomenon itself but rather on the biological behavior, stage, and therapeutic responsiveness of each individual tumor component. Therefore, management should be individualized according to the clinicopathological characteristics of each tumor.

In conclusion, this case represents a rare pulmonary collision tumor consisting of primary lung cancer and a metastatic lung tumor, as confirmed by preoperative CT findings. Histopathological and immunohistochemical analyses of resected specimens are essential for a definitive evaluation.

## Author Contributions

**Momoko Asami:** conceptualization, writing – original draft. **Shinya Katsumata:** writing – review and editing. **Hideaki Kojima:** writing – review and editing. **Nobuhiro Kado:** investigation, validation, writing – review and editing. **Yasuhisa Ohde:** writing – review and editing, supervision. **Mitsuhiro Isaka:** writing – review and editing. **Masahiro Endo:** writing – review and editing, investigation, validation. **Naoya Yokomakura:** writing – review and editing. **Takuya Kawata:** investigation, validation, writing – review and editing. **Hayato Konno:** writing – review and editing, supervision, conceptualization.

## Funding

The authors have nothing to report.

## Consent

Written informed consent was obtained from the patient for publication of this case report and any accompanying images.

## Conflicts of Interest

The authors declare no conflicts of interest.

## Data Availability

The data that support the findings of this study are available on request from the corresponding author. The data are not publicly available due to privacy or ethical restrictions.
